# Functions of music, focused on the context of music listening, and psychological well-being in late adolescence regarding gender differences

**DOI:** 10.3389/fpsyg.2023.1275818

**Published:** 2023-12-21

**Authors:** Katarina Habe, Snježana Dobrota, Ina Reić Ercegovac

**Affiliations:** ^1^Academy of Music, University of Ljubljana, Ljubljana, Slovenia; ^2^Faculty of Humanities and Social Sciences, University of Split, Split, Croatia

**Keywords:** functions of music, the context of music listening, psychological well-being, adolescence, students, gender differences

## Abstract

Late adolescences, as a developmentally challenging transitional period between childhood and adulthood, provides a number of pressures that impact well-being of youth. Among approaches for facilitating well-being, music is reported to be one of the most effective ones, which was especially proven during Covid-19 pandemic. Given the significance of music and music listening in late adolescence, our study sought to examine the relationship between psychological well-being and music listening among university students (*N* = 603; *N*female = 356, *N*male = 247) with a focus on the social, intrapersonal, and sociocultural context of music listening. The RESPECT music scale, the SPWB, and the PANAS were used to measure positive and negative affect as well as the six components of psychological well-being. The findings revealed that, while there were no gender differences in the sociocultural setting, females reported to listen to music more frequently than males in intrapersonal and social contexts. In two areas, female students rated their psychological well-being higher than male students: personal growth and positive relationships with others. They also reported experiencing positive and negative affect more frequently than men. Regression analyses revealed that the functions of music explained only a small amount of the variance in psychological well-being. Specifically, music listening in a social and sociocultural context significantly explained two aspects of psychological well-being: personal growth and positive relations with others. The intrapersonal context of music listening predicted a positive affect, while the social context predicted a negative affect. Our study highlights several implications of music listening in youth regarding gender either in everyday activities or in educational and clinical setting.

## Introduction

The use of simple, non-invasive stress-reduction techniques for facilitating well-being is becoming more and more popular (MacDonald, 2013). Music-related activities are thought to be particularly beneficial among those that encourage positive emotions because they are easily accessible and inexpensive ways to improve well-being (Dingle et al., 2018; Kappert et al., 2019; de Witte et al., 2020; Sheppard and Broughton, 2020). The socioemotional purposes of musical activities (e.g., listening to music) have historically been emphasized when discussing the psychological functions of music (Hargreaves and North, 1999; Schäfer et al., 2013). This includes a variety of purposes, including emotion regulation, social interactions, enjoyment and amusement, alleviation of worry and tension, and distraction (e.g., Toyoshima et al., 2011; Sanal and Gorsev, 2014; Schäfer et al., 2020; Schäfer and Eerola, 2020).

Particularly in late adolescence, when individuals face numerous developmental challenges, mechanisms for effective coping with mental health issues and for promoting psychological well-being are of a great importance (Frydenberg, 2018). The use of music as a facilitator of well-being is therefore especially relevant (Miranda, 2013; McFerran et al., 2019; Krause et al., 2021), because adolescents consume a significant amount of music (Bonneville-Roussy et al., 2013; Dingle et al., 2018) and “The meaning and importance of music to young people seems to be tied to their psychosocial development” (Laiho, 2004, p. 48). Lipson and Eisenberg (2018) state, that late adolescence is the period when psychological challenges are most likely to manifest. According to some studies, students report mental health issues at higher rates than either the general public (Stallman, 2010) or their peers who are not studying (Kovess-Masfety et al., 2016). Research indicates that a significant portion of university students are coping with mental health concerns, including emotional and psychological distress, anxiety and depression, and a higher risk of burnout (Baik et al., 2019; Backhaus et al., 2020). Adolescents’ mental health is influenced by a variety of factors, including effective coping mechanisms, positive mood, social support, self-esteem, high levels of perceived control over stressors, and opportunities for growth-promoting leisure activities (Lök et al., 2017; Basu and Banerjee, 2020; Nagy-Pénzes et al., 2020).

Based on the previous research findings that listening to music is one of the best ways to facilitate well-being in adolescence (Bland et al., 2012; McFerran et al., 2019; Miranda, 2019; Saarikallio et al., 2019; Agarwal et al., 2022), we set out to investigate how the social, intrapersonal, and sociocultural context of music listening is related to student’s well-being in late adolescence. In addition, gender differences were a topic we wanted to investigate because they have been found to have an important impact on how music contributes to psychological well-being (Liljeström et al., 2012). In the theoretical backgrounds we are going to first present studies in psychological well-being of adolescents, outline key functions of music in adolescence, and at the end focus on gender differences regarding the relationship between functions of music and psychological well-being.

### Psychological well-being of adolescents

Nowadays, rapidly increasing psychological distress is turning into a mental health crisis and leading to the emergence of mental diseases in students (Qiu et al., 2020). Emotionally charged states are prevalent in young people going through adolescence (Casey and Caudle, 2013) and stress is therefore a normal developmental phenomenon in that period (Shankar and Park, 2016; Wang et al., 2019). However, Covid-19 pandemic brought up additional stressors that affected well-being of university students (Dodd et al., 2021; Gestsdottir et al., 2021; Holm-Hadulla et al., 2021; Burns et al., 2022; Ebrahim et al., 2022; Liu et al., 2022; Ropret et al., 2023). The ability to control individuals’ emotions is in late adolescence still growing (Beauchaine, 2015; Hannesdóttir and Ollendick, 2017). The effective emotion regulation abilities may help reduce dysphoria – a state of feeling very unhappy, uneasy, or dissatisfied – which is a frequent attendant in students’ emotional experiencing (Berking and Wupperman, 2012; Every-Palmer et al., 2020).

The conventional psychological approach outlines the perspective that stress and its effects on well-being are mostly comprehended through a focus on the control of unfavorable consequences (Folkman and Moskowitz, 2000; Denovan and Macaskill, 2017), which does not adequately address the coping strategies. Therefore, since the start of the twenty-first century, the concept of well-being has drawn a lot of attention (Dodge et al., 2012; Coffey et al., 2015).

Psychological well-being consists of affective and cognitive components. The affective component refers to emotional experiences associated to one’s psychological condition, which can be positive or negative, and the cognitive component refers to a subjective assessment of well-being and life satisfaction (Musek, 2015). Based on the affective or cognitive approach, there are two fundamental models of psychological well-being; hedonic model – a model of subjective (emotional) well-being (well-being, satisfaction with life), and eudaimonic model – model of psychological well-being, which is broader and focuses on experiencing the meaning of life and gaining self-actualisation. Ryff (1989) highlighted several characteristics of eudemonic well-being, including autonomy, environmental mastery, positive interpersonal relationships, sense of purpose in life, potential realization, and self-acceptance. We employed a measure of eudemonic well-being and affect measurements for the current study since affect is a crucial part of emotional well-being.

Psychological well-being of university students has been widely explored in the recent years (Ivić, 2017; Baik et al., 2019; Lattie et al., 2019; Liu et al., 2019; Konrad, 2020; Dodd et al., 2021; Habe et al., 2021; Kohls et al., 2021; Saddique et al., 2021; Villani et al., 2021; Yıldırım and Tanrıverdi, 2021; Ropret et al., 2023). According to studies from several nations, the well-being and mental health of students appear to be under pressure (Backhaus et al., 2020; Dopmeijer, 2021). Douwes et al. (2023) report various factors that students mention as facilitators of their well-being, such as personal and university related factors to external factors beyond their educational institution. Psychological well-being in university students was reported to be related to optimism, health values, and religiousness (Burris et al., 2009), resilience and empathy (Vinayak and Judge, 2018), self-efficacy (Siddiqui, 2015), social support (Holliman et al., 2021) and mindfulness (Rehman et al., 2023).

### The functions of music in adolescence

According to Schäfer et al. (2013) listening to music serves a variety of functions and there is a heterogeneous image about the quantity and nature of musical functions because of the various theoretical viewpoints, methodologies, and samples. Furthermore, the fundamental parameters underlying these functions are still up for debate (Schäfer et al., 2013).

We based our study on Boer’s taxonomy of musical functions (Boer et al., 2013), because it was the most suitable one to address cross-cultural differences, which we investigated in our larger study. Boer (2009) made a distinction between two dimensions of music functions: (1) personal focus (self-regulation or emotional expression) vs. social and cultural activity, and (2) pleasure and affect (enjoyment or relaxation) vs. contemplation functions (construction of self-identity, values or inspiration).

In adolescence music serves a variety of functions. Laiho (2004) proposed a theoretical model of music’s psychological functions in adolescents. The model is a classification of the psychological objectives guiding and providing context for ordinary musical activity. It finds support and is based on the adolescent developmental task from a variety of studies conducted across other fields. The model of psychological functions of music in adolescence consists of four categories: interpersonal relationships, identity, agency, and emotional field, which represent different areas of psychological functions that engaging with music can support. Music has a significant impact on growth and mental health of adolescents by helping them to satisfy these psychological goals.

University students and youth frequently listen to music, especially on-line (Kotsopoulou and Hallam, 2010; Hu et al., 2021). The most frequent functions of music in younger adults are affect management and social connection (Groarke and Hogan, 2015, 2019; McFerran et al., 2019). Song lyrics play a significant role in achieving the two aforementioned dominant functions of music (Miranda, 2013). Teenagers can strongly relate to music since the lyrics of their favorite songs typically deal with topics that are significant to them (such as their values, self-perception, relationship challenges, rebellion against parents and other authorities, obtaining independence, etc.). They can also use music to relieve negative emotions like anger, grief, and disappointment (Lacourse et al., 2001; McFerran et al., 2019).

### The connection between music listening and well-being in late adolescence

Music activities have historically been proven to be one of the most effective ways for enhancing well-being (Gustavson et al., 2021). Moreover, music listening has demonstrated itself as a particularly potent avenue for enhancing the well-being of adolescents (Cabedo-Mas et al., 2021; Ferreri et al., 2021; Krause et al., 2021; Martín et al., 2021; Martínez-Castilla et al., 2021; Ribeiro et al., 2021; Vidas et al., 2021; Hansen, 2022; Feneberg et al., 2023). The research on music listening has been particularly productive in exploring the effects of environmental and personal characteristics on well-being (e.g., Greb et al., 2018, 2019).

Previous findings point to a possible relationship between young adults’ psychological health and music listening (Greitemeyer, 2009; Chin and Rickard, 2014; Carlson et al., 2015; Papinczak et al., 2015; Vuoskoski and Eerola, 2017). Mental health specialists, especially those working with young adults, have advised music listening as a coping skill due to its unique influence on the brain (Groarke et al., 2020). Research have shown that music has a beneficial effect on students’ academic performance, attention span, emotional control, and intellectual development (Hallam, 2010). Specifically, studies have shown that young individuals with sleep issues can benefit from listening to calming classical music (Papinczak et al., 2015). Adolescents’ ability to cope with stress has also been shown to be enhanced by music (Gilligan, 2000). According to a survey-based study conducted among university students in Canada, music increased their general happiness (Morinville et al., 2013).

### Gender differences in functions of music and psychological well-being

According to Buckman (2017), several experts hypothesize that the way men and women listen to music differs in neurological and psychological ways.

Regarding neurological differences, Koelsch argues that males and females experience music differently in their brains based on fMRI data (Koelsch et al., 2003). Koelsch et al. (2003) investigated gender differences in the functional organization of the brain for music processing. Results demonstrate that an electrophysiological correlate of music-syntactic processing is generated bilaterally in females, and with right hemispheric predominance in males. The authors concluded that gender differences for the analysis of auditory information are not restricted to processes in the linguistic domain such as syntax, semantics, and phonology. The physiological disparities in emotional control between men and women may be the cause of these musical behavior variances (Bradley et al., 2001). As indicated in various research from a psychophysiological standpoint, listening to music affects listeners’ emotional responses in a way that depends on their gender (Wuttke-Linnemann et al., 2019).

Considering psychosocial perspective in explaining gender differences, Sergeant and Himonides (2014) claim that differences in personality traits resulting from social norms cause males and females to experience music differently. Neuroticism, which is frequently stereotyped in females, tends to result in more intense experiences of music-related notions (Liljeström et al., 2012), and females show greater sensitivity to “aversive” musical stimuli (Nater et al., 2006). Music is also used more frequently among female listeners to fulfil their emotional needs (North et al., 2000; Upadhyay et al., 2017). Gender differences in music preferences rely on socialization into male hardness and feminine emotionality in accordance with gender norms (North and Hargreaves, 2007; Colley, 2008; Hargreaves et al., 2012; Herrera et al., 2018; Dobrota et al., 2019). According to the authors, socialization influences gender differences in ways that favor emotional coping in women and a more logical approach to coping with difficult circumstances in males (Adler and Harrison, 2004). However, according to Hallam et al. (2012), there are no gender differences in the ways that music affects psychological well-being while people are passively listening. In addition, Juslin (2005, p. 93) states that while gender differences may occur, they may not be important and that “listener judgments are only marginally affected by musical training, age, and gender of the listener,” a viewpoint that is shared by others (Gabrielsson and Juslin, 1996, 2003).

Results from Boer et al. (2012) demonstrate that music is used by women more frequently for regulating emotions, dancing, and cultural identity than by men, and that there are gender disparities in the functions of music. Significant gender disparities in background and family-bonding behaviors were also confirmed. Once more, female listeners utilize these features of including emotive and contemplative aspects more frequently than male listeners.

#### The present research

The present research and the presented results are part of larger study conducted with the objective of examining how functions of music, musical preferences, psychological well-being, and gender relate to one another across cultures (Habe et al., 2018; Dobrota et al., 2019). This specific study aimed to determine whether the functions of music, focused on the context of music listening (intrapersonal, social, sociocultural), affect the psychological well-being from both perspectives, hedonistic and eudemonic. University students, who may be considered as a population at risk from a developmental standpoint, were the subject of our study. Given the difficulties of transitioning to adulthood and the financial and material hardships typical of that stage of life, university students are a particularly vulnerable demographic for mental health issues in terms of developmental risk proneness (Auerbach et al., 2018; Husky et al., 2020), which was especially obvious during Covid-19 pandemic (Raj and Fatima, 2020; Habe et al., 2021).

Our purpose was to fill the gap in exploring functions of music beyond its affective dimension, which was highlighted in previous studies (see Boer et al., 2012). We particularly aimed to explore the social dimension in music listening context. Furthermore, we wanted to explore gender differences in the functions of music and psychological well-being. Since earlier research, based on a model by Boer et al. (2012), used a variety of basic functions of music (e.g., dancing, venting, social bonding with friends, etc.), the focus of the present study was the context of music listening; specifically, intrapersonal, social and sociocultural. Drawing on our own previous results and the results of other studies, we expected that the intrapersonal and social context of music listening would be more pronounced in females.

The following research questions guided the current study:

- Are there gender differences in functions of music, focused on the listening context (intrapersonal, social, sociocultural)?
- Are there gender differences in psychological and subjective well-being?
- Could functions of music predict psychological and subjective well-being, beyond gender?

## Methods

### Participants and procedure

The study involved 603 participants. To obtain the results presented in the present paper, data from *N* = 603 students from Croatian (59%) and Slovenian (41%) universities were analysed. The participants’ average age was M = 20.69 years (SD = 2.01). Regarding gender, 356 individuals reported being female (59%) and 247 reported being male (41%). None of them selected the option “other.” The students were mostly Caucasian, recruited through regular classes at different public universities and were studying humanities and social sciences (63.5%) or technical sciences (36.5%).

The questionnaires used were administered to the participants in university facilities during regular classes. After briefly explaining the research aims, the students were asked to participate in the research and to fill out the questionnaires, which took approximately 25 min.

The study was conducted in accordance with the Slovenian and Croatian Psychological Societies’ instructions, following the standards of the Ethics Committee of the University of Ljubljana (Slovenia) and the University of Split (Croatia). The written and informed consent was collected from each adult participant in accordance with the Declaration of Helsinki.

### Instruments

*The RESPECT music scale* (Boer et al., 2012) consists of 45 items for assessing ten basic functions of music (Boer et al., 2012; Dobrota et al., 2019). These ten functions can be grouped into three domains with regard to the context of music listening: intrapersonal, social, and sociocultural. The intrapersonal context includes the emotional functions of music, music for venting or reducing stress, the spirituality function, music as background, and music for focus enhancement. The social context includes music as a means for social binding with friends, dancing and expressing values. The sociocultural context includes political attitudes, social bonding within family and cultural identity reflected in music. The subjects were instructed to circle one number on a scale of 1 to 7 (1 = not at all, 7 = significantly) to assess the degree to which each item reflects their experience with music. Three domains had good internal consistencies; therefore, three total scores were formed by summing the items comprising each domain. The descriptive parameters are presented in Table 1.

**Table 1:** Descriptive parameters of measures (*N* = 603).

		Cronbach α	*M*	*SD*	Range	*n*	Skewness	Kurtosis
Context of music listening	Intrapersonal	0.89	88.74	19.34	23–133	19	−0.35	−0.23
	Social	0.91	74.06	17.56	20–105	15	−0.51	−0.13
	Sociocultural	0.87	32.93	12.41	11–77	11	0.65	0.30
Psychological well-being	Autonomy	0.66	32.38	4.92	12–42	7	−0.23	−0.16
	Self-acceptance	0.81	31.95	5.99	9–42	7	−0.69	0.67
	Positive relations with others	0.72	32.63	5.57	16–42	7	−0.56	−0.18
	Personal growth	0.68	33.38	4.88	14–42	7	−0.72	0.97
	Purpose in life	0.68	31.14	5.71	12–42	7	−0.47	−0.13
Affect	Positive affect	0.80	33.93	6.21	13–50	10	−0.14	−0.31
	Negative affect	0.81	26.08	7.10	10–47	10	−0.13	−0.69

*n*, number of items.

*The Scales of Psychological Well-Being* (SPWB) (Ryff, 1989; Ryff and Keyes, 1995) measure six dimensions of psychological well-being, including autonomy, self-acceptance, positive relations with others, environmental mastery, purpose in life and personal growth. The original version of the scale had 20 items for each subscale, but other shorter versions were later developed. In the present research, we used a version with a total of 42 items (Ryff and Keyes, 1995; Ryff, 2014), with seven items in each subscale. The participant’s task was to rate their level of agreement with each statement on a 6-point scale (1 – strongly disagree, 6 – strongly agree). Previous research on Croatian samples did not confirm the original factor structure. For example, Brajša Žganec et al. (2014) also used a 42-item version of the scale, but could not replicate the original structure and found that a shortened 30-item version (five items for each subscale) had better construct validity. The authors also found one higher-order factor and good reliability for one composite score. We, too, were unable to replicate the original structure, and the six-factor model showed poor goodness-of-fit parameters. We therefore examined the reliabilities of the six subscales and found very low reliability for the environmental mastery subscale, which was consequently omitted from further analyses. The other subscales had good internal consistencies (Table 1), and five composite results were formed by summing the answers for the items comprising each subscale. The results on the five subscales were significantly interrelated, with correlation coefficients ranging from 0.26 between autonomy and positive relations with others, to 0.59 between purpose in life and self-acceptance.

*The Positive and Negative Affect Schedule* (PANAS) (Watson et al., 1988) is a self-reported affective scale that includes 10 positive and 10 negative emotions. The participant’s task was to rate how frequently, on a scale of 1 to 5, they generally felt in the manner indicated. The internal consistency of both measures was high (Table 1). The correlation between positive and negative affect was significantly negative (*r* = −0.31).

### Data analyses

Since all the variables’ skewness ranged from −1 to +1 with the exception of political opinions, where it was 1.34, parametric tests were utilized for the analyses. Kurtosis, on the other hand, varied from −1 to +1, with the exception of political sentiments, where it was 1.35. Following the suggested cut-off values, there was no discernible skewness or kurtosis in the variables that were included (Trochim and Donnelly, 2006; Gravetter and Wallnau, 2014). Consequently, the *t*-test for independent samples was employed to address the gender disparities in the functions of music and psychological well-being. The relationship between gender, musical functions, and psychological well-being was examined using Pearson correlation coefficients, and the role of musical functions in influencing gender-based differences in psychological well-being was examined using hierarchical regression analysis. In correlational and regression analyses gender was coded binary – 1 males, 2 females.

## Results

Table 2 presents gender differences in the context of music listening, affect and psychological well-being. The female participants had higher results in the intrapersonal and social context of music listening, positive and negative affect, positive relations with others, and personal growth.

**Table 2:** Gender differences in the context of music listening and psychological well-being.

	M_f_	SD_f_	M_m_	SD_m_	*t* (df = 601)	Cohen’s *d*
Intrapersonal context of music listening	4.92	0.94	4.31	1.01	7.55**	0.63
Social context of music listening	6.33	1.01	5.66	1.10	7.79**	0.63
Sociocultural context of music listening	3.04	1.07	2.96	1.21	0.90	–
Positive affect	3.45	0.59	3.31	0.63	2.63**	0.21
Negative affect	2.67	0.68	2.52	0.73	2.71**	0.21
Autonomy	4.61	0.70	4.65	0.72	−0.69	–
Self-acceptance	4.60	0.83	4.52	0.89	1.07	–
Positive relations with others	4.77	0.76	4.50	0.81	4.23**	0.34
Personal growth	4.88	0.62	4.60	0.77	4.91**	0.40
Purpose in life	4.48	0.78	4.40	0.87	1.15	–

*p < 0.01; **p < 0.01; M_m_, Mean for males; M_f_, Mean for females; SD_m_, Standard deviation for males; SD_f_, Standard deviation for females.

Table 3 presents a correlation matrix of all of the variables included in the study. Positive affect was positively related to all facets of psychological well-being, while negative affect was negatively related to autonomy, self-acceptance and purpose in life. The intrapersonal context of music listening was positively related to both aspects of affect, while there were no significant correlations with aspects of well-being. The social function of music was also related to both aspects of affect, and to positive relations with others and personal growth. The sociocultural context of music listening was related to negative affect and negatively related to positive relations with others.

**Table 3:** Correlation matrix of variables included in the study (values, context of music listening and psychological well-being, *N* = 603).

	1	2	3	4	5	6	7	8	9	10
1 Gender										
2 Intrapersonal context of music listening	0.29**									
3 Social context of music listening	0.30**	0.66**								
4 Sociocultural context of music listening	−0.04	0.40**	0.42**							
5 Positive affect	0.11**	0.19**	0.08*	0.01						
6 Negative affect	0.11**	0.14**	0.22**	0.15**	−0.31**					
7 Autonomy	−0.03	−0.04	−0.01	−0.07	0.16**	−0.20**				
8 Self-acceptance	0.04	−0.02	0.02	0.02	0.22**	−0.18**	0.43**			
9 Positive relations with others	0.17**	0.05	0.13**	−0.10*	0.10*	−0.04	0.27**	0.44**		
10 Personal growth	0.20**	0.07	0.14**	−0.04	0.21**	−0.02	0.34**	0.47**	0.41**	
11 Purpose in life	0.05	0.03	−0.00	−0.04	0.20**	−0.08*	0.36**	0.59**	0.42**	0.50**

*p < 0.05; **p < 0.01.

Two steps were taken in the regression analyses shown in Table 4. Given that *t*-tests revealed a significant difference between the genders in four categories, gender was taken into account in the initial phase of all analyses. In analyses using positive/negative affect, positive relationships with others, and personal growth as criteria, gender was discovered to be a significant predictor. The second phase introduced the functions of music. The findings revealed that just two aspects of psychological well-being – personal growth and pleasant relationships with others – are significantly predicted by the context of music listening. The benefits were, however, fairly minimal, and the ways in which music serves only partially account for the variation in the criteria. Positive relations with others could be partially explained by gender, the social function of music, and the sociocultural context of music listening (negatively). Gender and the social function of music were significant predictors of personal growth. In analyses with affect as criteria, gender was a significant predictor in the first step, but introducing the functions of music in the equation made gender become insignificant. Positive affect could be predicted by the intrapersonal function of music, while the social function of music explained a significant portion of the variance of negative affect. It is possible that the functions of music partially mediated the effect of gender on positive and negative affect.

**Table 4:** Hierarchical regression analyses with psychological well-being and affect as criteria.

	Autonomy	Self-acceptance	Positive relations with others	Personal growth	Purpose in life	Positive affect	Negative affect
Step 1							
Gender	−0.03	0.04	0.17	0.20	0.05	0.11	0.11
*R (R^2^)*	0.03 (0.001)	0.04 (0.002)	0.17 (0.03)	0.19 (0.04)	0.05 (0.002)	0.11 (0.01)	0.11 (0.01)
*F (df)*	0.47 (1,601)	1.15 (1,601)	17.92** (1,601)	24.11** (1,601)	1.32 (1,601)	6.91** (1,601)	7.33** (1,601)
Step 2							
Gender	−0.04	0.05	0.12**	0.16**	0.04	0.06	0.07
Intrapersonal context of music listening	−0.03	−0.07	−0.05	−0.04	0.06	0.24**	−0.03
Social context of music listening	0.06	0.04	0.19**	0.15**	−0.03	−0.06	0.18**
Sociocultural context of music listening	−0.08	0.03	−0.16**	−0.08	−0.05	−0.06	0.09
*R (R^2^)*	0.09 (0.01)	0.07 (0.005)	0.24 (0.06)	0.23 (0.05)	0.08 (0.005)	0.21 (0.04)	0.23 (0.05)
*ΔR^2^*			0.03*	0.01*		0.03*	0.04*
*F (df)*	1.14 (4,598)	0.75 (4,598)	9.42** (4,598)	8.42** (4,598)	0.85 (4,598)	6.87** (4,598)	8.69** (4,598)

*p < 0.05; **p < 0.01.

## Discussion

The present research explored the relationship between gender, functions of music focused on the context of music listening, affect and psychological well-being in university students in late adolescence.

In order to respond to our initial research question, we first intend to address the discovered gender inequalities in the context of music listening and well-being. Female students reported to listen to music more in intrapersonal and social contexts than male students do, while there were no appreciable differences in the sociocultural context. These findings indicate that women, more than men, use music for fulfilling a wide range of needs both in intrapersonal and social contexts. This is consistent with a number of earlier studies that found that men and women listen to music differently (Buckman, 2017) and that personality differences brought on by social norms affect how men and women experience music (Sergeant and Himonides, 2014), with females reporting more intense experiences of music-related notions (Liljeström et al., 2012). Our results therefore converge with several studies confirming that music listening influences listeners’ emotional reactions in a gender-dependent manner (North et al., 2000; Upadhyay et al., 2017; Wuttke-Linnemann et al., 2019). Gender differences in music listening can be explained also from the physiological perspective; Koelsch et al. (2003) claim that males and females respond to music in different ways neurologically, and Bradley et al. (2001) argue that gender differences in affect proneness and physiological disparities in emotional regulation may be the basis for these variations in musical behavior.

In response to the second research question about gender differences in well-being, we were able to observe that female students reported more positive and negative affect, as well as personal growth and positive relationships with others than male students did. Although previous findings on gender differences in emotional well-being (affect) are not unambiguous, some studies found no gender differences across PANAS scales (Crawford and Henry, 2004; Merz et al., 2013; Sanmartin et al., 2018). However, other studies (Inglehart, 2002; Diener and Ryan, 2014; Zuckerman et al., 2017) have demonstrated that women have a greater range of positive and negative affect than men. According to previous studies (Yeo et al., 2007; Pascual et al., 2012; Wrobel et al., 2019), after the turbulent period of early and middle adolescence, when girls typically experience fewer positive emotions than boys, women show higher frequency and higher intensity of all emotions, regardless of their valence. It is possible that gender differences in affect are the result of gender-role socialization into female emotionality. In addition to temperamental differences (Else-Quest et al., 2006), which serve as a foundation for later gender differences in emotional expressiveness, Brody (2009) pointed out that emotional expressiveness is encouraged more in girls than in boys during socialization, which results in gender differences in emotional expression throughout life. Regarding psychological well-being our results, which show that female students report more pronounced positive relations with others and express higher personal growth than male students, converge with several previous studies (Brajša Žganec et al., 2014; Matud et al., 2014; Matud and García, 2019). The findings can be explained in the context of females generally being encouraged to nurture good relationships with others through the socialization process; specifically, girls are usually expected to be more empathic and considerate towards others and their good relationships with others are probably reinforced, thus resulting in the development of more skills for maintaining positive relations with others (Quatman and Watson, 2001; Weisberg et al., 2011). However, in the literature on gender differences in psychological well-being mixed findings can be observed, which are probably the result of different conceptualizations and measures used for assessing well-being, as well as the use of heterogeneous samples. According to Dreger et al.’s (2016) analysis of data from the third round of the European Quality of Life Survey (2011–12), women often experience lower mental well-being than men. In contrast, studies utilizing the PWB measures (Ryff, 1989; Ryff and Keyes, 1995) in a sample of adolescent high school students indicated no gender differences in psychological well-being (apart from self-acceptance) (Visani et al., 2011). In a study conducted by Matud et al. (2014) on a sample of adults, men scored higher on autonomy and self-acceptance, while women scored higher, similarly to our findings, on personal growth and good relationships with others.

Our key research question was if the functions of music, focused on the listening context, can predict psychological and emotional well-being, beyond gender. We therefore tried to explore the context of music listening in late adolescence relevant to several aspects of psychological well-being, according to the conceptualisation of Ryff (Ryff, 1989; Ryff and Keyes, 1995). The results pointed to weak connections between well-being and the functions of music. Specifically, the social function of music was predictive for most facets of well-being, including positive relations with others and personal growth, and to negative affect. Adolescents who listen to music more in a social context (for social bonding with friends, expressing values, dancing) assessed higher personal growth and positive relations with others, but also negative affect. Regarding affect, it can be assumed that students use music for negative emotion regulation in a social context, i.e., with their peers and friends who share the same concerns, difficulties or other similar characteristics typical of the student population. It may be that negative emotions induce them to listen to music with their friends as a way of relaxing and reducing stress, or as a way of dealing with the psychologically or emotionally challenging situations they face (Saarikallio and Erkkilä, 2007; Saarikallio, 2011). Furthermore, music listening in a social context may reflect the need for fostering social relations, which are very important in this life period. The need for relatedness is one of the basic psychological needs (Deci and Ryan, 2000). Music listening in a social context can fulfil this need and sharing the experience of music listening can connect young people. Since music listening in a social context can successfully fulfil a young person’s psychological need, it may also be relevant for personal growth. Further research should explore whether psychological needs could explain the relationship between different ways of listening to music (and the importance of music during adolescence and emerging adulthood) as well as aspects of psychological well-being.

The intrapersonal function of music was shown to be an important predictor only for experiencing positive affect, while relations with other aspects of well-being were not significant. Young people who listen to music more in an intrapersonal context, for emotional regulation, for enhancing personal focus or as background, expressed higher positive affect. Our findings are consistent with those of the Boer et al. (2012), who found that students from six different cultures ranked emotional expression and venting as two of the three most crucial functions of music. Additionally, they discovered very minor cross-cultural differences in each of the intrapersonal functions of music, confirming its significance and cultural universality.

If we sum up, our findings about the ways in which music helps adolescents regulate their emotions and connect with others are consistent with the findings of several earlier studies (Groarke and Hogan, 2015, 2019; McFerran et al., 2019). It is also worth mentioning that correlation analyses showed that both positive and negative emotions are positively related to the intrapersonal function of music, regardless of the negative correlation between positive and negative affect. These results may point to the conclusion that regardless of the valence of emotions, music can be a tool for regulating different emotional experiences.

Prior to drawing a conclusion, it is important to address a few of the research’s limitations. Firstly, the measure of psychological well-being revealed worse than anticipated reliability parameters. The reliability coefficient for the environmental mastery subscale was too low, which is why we did not use this subscale in further analyses. Furthermore, internal consistencies for the autonomy, personal growth and purpose in life subscales were somewhat lower, although still acceptable. Secondly, all the results were obtained via questionnaire data/self-reports, which can lead to subjectivity of the results.

Despite its limitations, the study’s findings contribute to the field of research on the connection between music and well-being by highlighting important differences between men and women in terms of their preferred musical environments as well as the association between musical listening in an intrapersonal or social setting and specific psychological and emotional well-being factors. In contrast to the vast majority of studies conducted to date, which largely focused on gender differences in the affective functions of music (see Boer et al., 2012), with our study, we filled the gap in studying differences between genders in functions of music relating to social and cultural context.

The study discusses a number of applications, emphasizing the value of adolescent music listeners’ social contexts. It demonstrates how crucial music listening is to adolescents’ well-being. Our findings support the notion that music listening during late adolescence might be used to promote well-being, particularly in women. In more concrete terms, listening to music while adolescent well-being is being regulated can be utilized as a preventive strategy as well as an intervention. Additionally, it’s critical to recognize gender disparities in listening contexts, as these distinctions may help us organize educational or therapeutic music interventions for male or female students differently.

These findings may provide several implications, for both listeners and health professionals, when considering how music listening can be used as a self-administered tool for coping with everyday stressors. Regarding gender differences, we could presume, that especially in female students, music listening could be promoted as an effective strategy for coping with stress and for facilitating well-being, especially for facilitating self-growth and building positive relationships with others in university students. Moreover, receptive music therapy could be recommended as one of the effective approaches in dealing with mental health challenges of adolescents, especially female students. Last but not least, our results reveal the importance of social context of music listening for promoting psychological well-being in university students.

In the future research it would be advisable to delve deeper into investigating the social functions of music listening. Moreover, it would be valuable to develop and validate a model for promoting well-being in university students through music listening in varied music listening contexts.

## Data availability statement

The raw data supporting the conclusions of this article will be made available by the authors, without undue reservation.

## Ethics statement

The studies involving humans were approved by Komisija za etiko Filozofske fakultete (KEFF). The studies were conducted in accordance with the local legislation and institutional requirements. The participants provided their written informed consent to participate in this study.

## Author contributions

KH: Conceptualization, Investigation, Resources, Writing – original draft, Data curation. SD: Investigation, Resources, Writing – review & editing. IR: Methodology, Validation, Writing – review & editing.
